# Differential Analysis of the Secretome of WRL68 Cells Infected with the Chikungunya Virus

**DOI:** 10.1371/journal.pone.0129033

**Published:** 2015-06-17

**Authors:** Christina Li-Ping Thio, Rohana Yusof, Ali Ashrafzadeh, Syareena Bahari, Puteri Shafinaz Abdul-Rahman, Saiful Anuar Karsani

**Affiliations:** 1 Institute of Biological Sciences, Faculty of Science, University of Malaya, 50603, Kuala Lumpur, Malaysia; 2 Department of Molecular Medicine, Faculty of Medicine, University of Malaya, 50603, Kuala Lumpur, Malaysia; 3 University of Malaya Centre for Proteomics Research (UMCPR), Medical Biotechnology Laboratory, Faculty of Medicine, University of Malaya, 50603, Kuala Lumpur, Malaysia; 4 Drug Design and Development Research Group (DDDRG), University of Malaya, 50603, Kuala Lumpur, Malaysia; 5 Medical Biotechnology Laboratory, Faculty of Medicine, University of Malaya, 50603, Kuala Lumpur, Malaysia; SRI International, UNITED STATES

## Abstract

The Chikungunya virus (CHIKV) is an arthropod borne virus. In the last 50 years, it has been the cause of numerous outbreaks in tropical and temperate regions, worldwide. There is limited understanding regarding the underlying molecular mechanisms involved in CHIKV replication and how the virus interacts with its host. In the present study, comparative proteomics was used to identify secreted host proteins that changed in abundance in response to early CHIKV infection. Two-dimensional gel electrophoresis was used to analyse and compare the secretome profiles of WRL-68 cells infected with CHIKV against mock control WRL-68 cells. The analysis identified 25 regulated proteins in CHIKV infected cells. STRING network analysis was then used to predict biological processes that may be affected by these proteins. The processes predicted to be affected include signal transduction, cellular component and extracellular matrix (ECM) organization, regulation of cytokine stimulus and immune response. These results provide an initial view of CHIKV may affect the secretome of infected cells during early infection. The results presented here will compliment earlier results from the study of late host response. However, functional characterization will be necessary to further enhance our understanding of the roles played by these proteins in the early stages of CHIKV infection in humans.

## Introduction

Previously a non-fatal and relatively benign disease, chikungunya (CHIK) has emerged as a potential global threat. This is evidenced by sudden outbreaks of unprecedented magnitude over the past decade, with greater morbidity seen in each successive outbreak. Since its first appearance in 1953, many countries have reported its re-emergence, including Malaysia, Indonesia, Thailand, India and the Réunion Island, with more than 7 million reported cases to date. Recent epidemiological documentation provided further evidence of the spread of CHIK infection to temperate countries such as Italy, Australia and the United States, where sporadic outbreaks have been reported [1].

This disease is caused by the Chikungunya virus (CHIKV). The virus belongs to the genus *Alphavirus* and family *Togaviridae* [2]. CHIKV is transmitted by the same vectors responsible for the spread of the dengue virus, namely *Aedes aegypti* and *Aedes albopictus* mosquitos. Infection with CHIKV causes an illness with the following symptoms—fever, rash and debilitating arthralgia. These symptoms may remain for years. In more recent cases however, an increase in atypical clinical symptoms such as neurological and cardiovascular complications has been observed [3]. Deaths attributed to complications of this disease are no longer unheard of, and the fatality rate is now estimated to be 1:1000 cases [4]. Moreover, most surviving patients are often incapacitated by recurring polyarthralgia that persists for years. Considering these factors, the epidemiological and socioeconomic burden brought about by this disease is a great cause for concern. Treatment is palliative and no effective antiviral drug or vaccine is currently available. Given the lack of preventive or therapeutic measures along with the recurring emergence and rapid spread of infection, CHIKV is now considered a potential global health problem.

Despite extensive research over the past several years, much remain unknown about the biology and mechanisms behind CHIKV pathogenesis. To unravel and comprehend key aspects of the infection, it is important to first grasp the mechanisms by which the virus interacts with its human host, and how the human host responds to the foreign pathogen. We have previously characterized the whole cell proteome of CHIKV infected host cells [5]. Here, we have extended the analysis to the secretome of infected host cells.

The secretome represents the entire complement of secreted proteins. Various mechanisms are involved in the secretion of these proteins. They include classical secretion through the migration of vesicles from the endoplasmic reticulum to the Golgi apparatus, through non-classical mechanisms that are vesicle-mediated, and also via the shedding of proteins from the surface of living cells. Approximately 10% of the human genome encodes for the secretome. The secretome profile may reflect the different biological/physiological conditions within a cell. Thus, potentially the secretome can be a source for biomarkers and drug targets. Apart from that, an understanding of how the secretome changes under different situations may lead to an understanding of occurrences within the cell. Given the scarcity of knowledge on the association between CHIKV and its human host in general, and the promising results obtained in other studies using proteomic approaches as the tool of study, it is therefore of great interest to look into changes in global protein profiles of host cells during CHIKV infection, particularly the secretome.

## Materials and Methods

### Preparation of the secretome

#### Cell lines

All cell lines were originally purchased from the ATCC collection. Cell lines used in this study were WRL-68 human hepatic cells, a HeLa derivative cell line (ATCC Cat No. CL-48), Vero cells (ATCC Cat. No. CCL-81), and C6/36 Aedes albopictus cells (ATCC Cat. No. CRL-1660). Culture and maintenance of cell lines were performed as previously described [5].

#### Antibodies

The antibodies used for indirect immunofluorescence assay (IIFA) and immunostaining by flow cytometry were as previously described [5]. The anti-CHIK E2 monoclonal antibody (mAb) 3E4 were a kind gift from Dr. Philippe Desprès from the Pasteur Institute of France and FITC-conjugated goat anti-mouse IgG secondary antibody (Novus Biologicals, Littleton, CO). The primary antibody used for Western blot were mouse mAb to GTP-binding nuclear protein ran (RAN). The secondary antibody used was horseradish peroxidise (HRP)-conjugated goat anti-mouse IgG. The antibodies used for western blotting were purchased from Santa Cruz Biotechnology, Santa Cruz, CA.

#### Virus stock propagation and titration

CHIK/06/08 clinical isolate of the ECSA genotype was propagated, and virus titer determined as previously described [5].

#### Infection of WRL-68 cells with CHIKV

The infection and determination of optimal multiplicity of infection (MOI) and time-point for early infection of WRL-68 cells with CHIKV was performed as previously described [5] with modifications. Briefly, WRL-68 cells were seeded at a density of 4.5 × 10^6^ cells in 75cm^2^ culture flasks overnight at 37°C. Cells were then infected with CHIKV in serum-free DMEM medium at the optimized MOI and incubated for 2 hours at 37°C with gentle agitation at 15 minute intervals. Viral inoculum was then removed and the cells were washed extensively with serum-free medium to remove traces of FBS from the virus supernatant. Infected cells were further incubated at the optimized time-point in serum-free DMEM medium. As a control, mock infected cells were cultured in parallel. Confirmation of infection and quantification of percentage CHIKV infection and cell death was performed as previously described [5].

#### Cell proliferation assay

The effect of serum deprivation on WRL-68 cell proliferation was determined using MTS cell viability assay (Promega, Madison, WI). Briefly, cells were seeded at a density of 1.0 × 10^4^ cells/well in a 96-well dish overnight. This was followed by extensive washing with serum-free DMEM medium and incubation in either DMEM growth medium or serum-free DMEM medium. Twenty μl of CellTiter 96 Aqueous One Solution Cell Proliferation reagent was then added to the cells, followed by four hours incubation at 37°C. Absorbance was then read at 490nm and a cell viability graph was plotted based on the data from three independent experiments. All data were presented as mean ± SD.

#### Dead-cell protease release assay

Quantification of cellular disruption was determined using CytoTox-Glo Cytotoxicity assay (Promega, Madison, WI) according to the manufacturer’s instructions. This was performed to assess the effect of serum depravation on membrane integrity. Briefly, WRL-68 cells were seeded at a density of 1.0 × 10^4^ cells/well in 96-well dish overnight. This was followed by extensive washing with serum-free DMEM medium and incubation in either DMEM growth medium or serum-free DMEM medium at the optimized incubation time (24 hours) to determine the effect of serum deprivation on cell membrane integrity.

The effect of CHIKV-infection at the optimized MOI (MOI 5.0) on WRL-68 cellular disruption was evaluated concomitantly by infecting the cells for 2 hours with CHIKV, followed by extensive washing with serum-free DMEM medium and further incubation for the optimized time point (24 hours). The relative percentage of cell lysis was calculated based on the maximum cellular disruption obtained from cells treated with lysis reagent. All data were presented as mean ± SD.

#### Collection of secretome

Serum-free DMEM medium of CHIKV-infected and control WRL-68 cells were collected in 50 ml centrifuge tubes and centrifuged at 3,000×g for five minutes to remove dead cells and cellular debris. The resulting supernatants were filtered through 0.22 μm syringe filters and further concentrated using Vivaspin 20 centrifugal concentrator with a 3kDa molecular weight cut-off membrane (Sartorius Stedim Biotech, Germany) and stored at -80°C.

#### Sample clean-up

Protein samples were desalted and concentrated using 2-D Clean-Up kit according to the manufacturer’s instructions (GE Healthcare, Uppsala, Sweden).

### Comparative proteomic analysis

#### Two-dimensional gel electrophoresis (2DE)

Protein concentration was determined by Bradford assay (BioRad, Philadelphia, PA, USA). A total of 40μg (for analytical gels) or 80μg (for preparative gels for MS analysis) of protein was used for each 2DE gel. Prior to 2DE, sample clean-up was performed using the 2D clean-up kit (GE Healthcare, Uppsala, Sweden). Proteins were then reconstituted in rehydration buffer (250μL, 7M urea, 2M thiourea, 2% [v/v] IPG buffer pH 3–10, 2% [w/v] CHAPS and bromophenol blue [trace]). Sample application by in-gel rehydration was then performed (13cm pH 3–10 linear IPG strips). This was followed by first dimension isoelectric focusing on an Ettan IPGphor III system (GE Healthcare, Uppsala, Sweden) using the following protocol: step and hold at 500V for 0.6kVh, gradient to 1000V for 1.0kVh, gradient to 8000V for 12.0kVh, step and hold at 8000V for 16.0kVh.

Following the first dimension separation of proteins, strips were placed in equilibration buffer containing 6 M Urea, 75 mM Tris-HCl pH 8.8, 29.3% Glycerol, 2% SDS, 0.002% Bromophenol Blue with 1% DTT for 15 min. This was followed by incubation in the same equilibration buffer containing 2.5% iodoacetamide for a further 15 min. Second dimension separation of proteins was then performed on 12.5% SDS-PAGE homogenous gels at 50 V for 30 min, and 500 V until the bromophenol blue dye reached the bottom edge of the gel.

Visualization of protein spots were performed using protocols described in the PlusOne Silver staining kit (GE Healthcare, Uppsala, Sweden). For analytical gels, the complete protocol was followed. A modified protocol was used for preparative gels where glutaraldehyde was not included in the sensitization step and formaldehyde not included in the silver reaction step [6].

#### Gel image analysis

Gels were scanned with ImageScanner III and analyzed using ImageMaster 2D Platinum v7.0 software (GE Healthcare, Uppsala, Sweden). The analysis was performed as previously described [5].

#### Tryptic digestion

Following 2DE, spots of interest were cut and in-gel digestion performed with trypsin (Promega) for mass spectrometric identification according to published protocols [7–9].

#### MALDI-TOF/TOF mass spectrometry analysis and database searching

Protein identification was performed as previously described using a MALDI-TOF mass spectrometer (ABI 4800 Plus, Applied Biosystems, Foster City, CA) [10, 11].

#### LC-Chip-MS/MS Q-TOF label-free quantification

All instruments and software used for mass spectrometry are from Agilent (Agilent, Santa Clara, CA, USA) unless otherwise stated. For label-free quantification, 50 μg protein from serum free culture media were subjected to in solution tryptic digestion. Digested samples were cleaned using Zip Tip (Millipore) following which the samples were lyophilized. The peptides from treated and untreated samples were reconstituted in 10μL of the first LC mobile phase (0.1% formic acid). To perform label-free relative quantitation, the samples of treated and untreated groups were analyzed over four technical replicates. Peptide separation was performed using a Nano-LC 1260 directly connected to an Accurate Mass Q-TOF 6550 with a Chip-Cube interface Nano-ESI ion source. In the chip column, peptides were first enriched using an enrichment column before being separated on a separation column (C18 reverse phase, 300Å, 150mm, 5 μm) with a 5–80% gradient of solvent B (0.1% formic acid in acetonitrile) for 24 min with a flow rate of 0.4 μL/min. Each mass data acquisition (8 spectra per second from 200–3000m/z) was followed by collision induced dissociation of the twenty most intense ions. MS/MS data were then acquired within the 50–3200m/z range. Acquired MS/MS data was then searched against the Swiss Prot human database to identify proteins.

The Spectrum Mill software was set to search the MS/MS acquired data against the Swiss Prot human database. Mass-tolerance of precursor and product ions was set to ±20 and ±50 ppm respectively. As iodoacetamide was used for alkylation during sample preparation, carbamidomethylation was specified as a fixed modification and oxidized methionine as a variable modification. The precursor mass shift was set between -18 Da to 177 Da to take into consideration variable modifications such as the presence of sodium and potassium adducts. Proteins that shared at least one peptide were grouped together. Identified proteins were then filtered to achieve a false discovery rate (FDR) of <1% for the peptide-spectrum matches.

For label-free relative quantitation, differentially expressed proteins were assessed based on the regulation of the peptides. Protein lists of treated and untreated groups were exported to the Mass Profiler Professional (MPP) software for statistical data analysis. Data analysis was carried out based on the total spectra intensity of the proteins which were considered as entities in MPP. The baseline of the spectra was adjusted to the median across all samples. The entities were then filtered based on their frequency of occurrence across at least all replicates of one treatment. Unpaired sample T-test was performed with the P<0.05. Benjamini-Hochberg corrected P-values were calculated to overcome the problem of multiple test analysis (false discovery). Regulated proteins with ≥ 1.5 fold change were then considered as regulated proteins.

#### *In Silico* analysis

In all bioinformatics analysis, canonical sequences of identified proteins were used.

For the prediction of signal peptide cleavage sites, the SignalP 4.0 server (http://www.cbs.dtu.dk/services/SignalP/) was used. A D-score exceeding 0.450 suggested the presence of a signal peptide within a protein sequence [12].

For the analysis of proteins not predicted to possess a signal peptide, the SecretomeP 2.0 server (http://www.cbs.dtu.dk/services/SecretomeP/) was used to predict proteins that may be secreted via a non-classical secretion pathway. A protein with N-N score above or equal to a threshold value of 0.500 was predicted to be non-classically secreted [13].

The presence of exosomes and apoptotic blebs may result in the shedding and secretion of transmembrane proteins into the extracellular. To identify the presence of transmembrane plasma protein features, identified proteins were analyzed using the TMHMM v2.0 server (http://www.cbs.dtu.dk/services/TMHMM-2.0/). A predicted transmembrane plasma protein were those with an expected number of amino acids in transmembrane helixes (ExpAA) exceeding 18.000 [14–16].

To be categorized as “secreted”, a protein must fulfil at least one of the following criteria: a signal peptide was contained within the protein according to SignalP, SecretomeP predicts that it is a non-classically secreted protein or it has a transmembrane helix according to TMHMM.

To predict protein-protein interactions, The STRING (Search Tool for the Retrieval of Interacting Genes/Proteins) database v9.0 (http://www.string-db.org/) was used. The search parameters used were as previously described [5]. Proteins were represented as nodes and their functional links were defined by solid lines. The thickness of the lines signifies the level of confidence of the reported association. Twenty additional interacting proteins were added to the interaction map in an attempt to draw up a more global view of biological processes that may be affected during CHIKV infection. The protein names and gene symbols used in the networks are listed in **S1 Table. Protein names and gene symbols used in the secretome network.**

#### Real-time PCR (RTPCR)

RTPCR and the ensuing statistical analysis was performed as previously described [5].

#### Western blotting

Western blotting was performed essentially as previously described [5]. The only exception was that the initial protein separation was performed in two dimensions.

## Results

### Preparation of the secretome

#### Effect of serum depravation on cell viability

**Fig 1:**
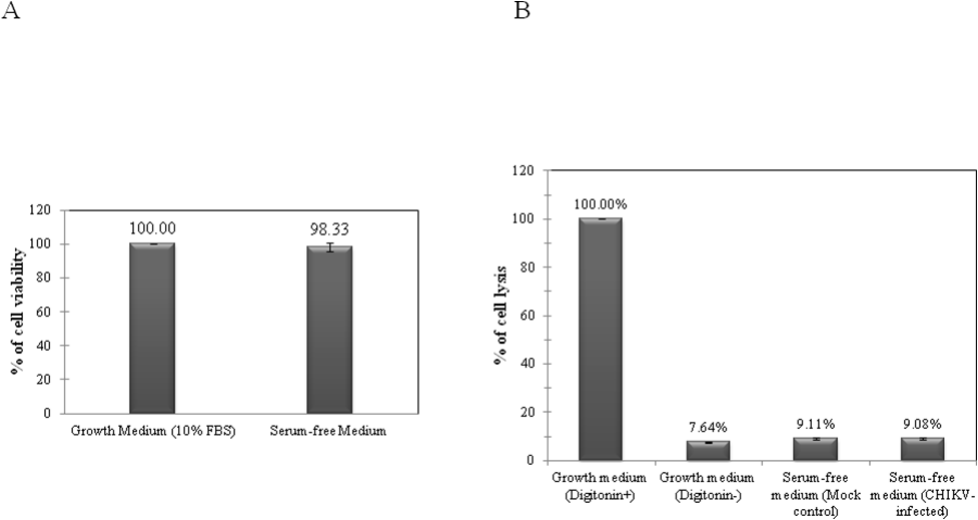
Relative cell viability (A) and percentage of cell lysis (B) of WRL-68 cells grown in DMEM growth medium (10% FBS) and serum-free medium for 24 hours. A: Cell viability was normalised to 100%, and the relative viability of serum-starved cells was determined. At 24 hours incubation, the absence of serum did not significantly reduce cell viability as percentage of viability was above 98%. Data presented are representative of three independent experiments and the error bars represent standard deviation. B: Percentage of cell lysis for digitonin-lysed cells (positive control) was normalised to 100%. The absence of serum in the culture medium was found to have no significant effect on cellular disruption. Similarly, WRL-68 cells infected with CHIKV at the MOI of 5.0 showed similar percentage as the mock control cells. Data presented are representative of three independent experiments and the error bars represent standard deviation.

MTS was used to investigate the viability of serum-starved WRL-68 cells. Fig 1A shows the relative viability of cells grown in normal DMEM medium (10% FBS) and serum-free medium where no significant difference in cell viability was observed.

#### Effect of serum deprivation and CHIKV infection on cell membrane integrity

Fig 1B shows the relative percentage of cell lysis between uninfected WRL-68 cells incubated in DMEM growth medium, serum-free medium and CHIKV-infected cells (MOI of 5.0) incubated in serum-free medium for 24 hours. Cells lysed with digitonin were used as positive control, normalized to 100%. A low percentage of cellular disruption (7.64% ± 0.29) was observed in cells cultured in growth medium. Only a slight increase in the percentage of lysed cells was detected in serum-free cultured cells (9.11% ± 0.46) and CHIKV-infected cells (9.08% ± 0.42), as compared to cells incubated in growth medium.

### Comparative proteomic analysis

#### Two-dimensional gel electrophoresis (2DE)

**Fig 2:**
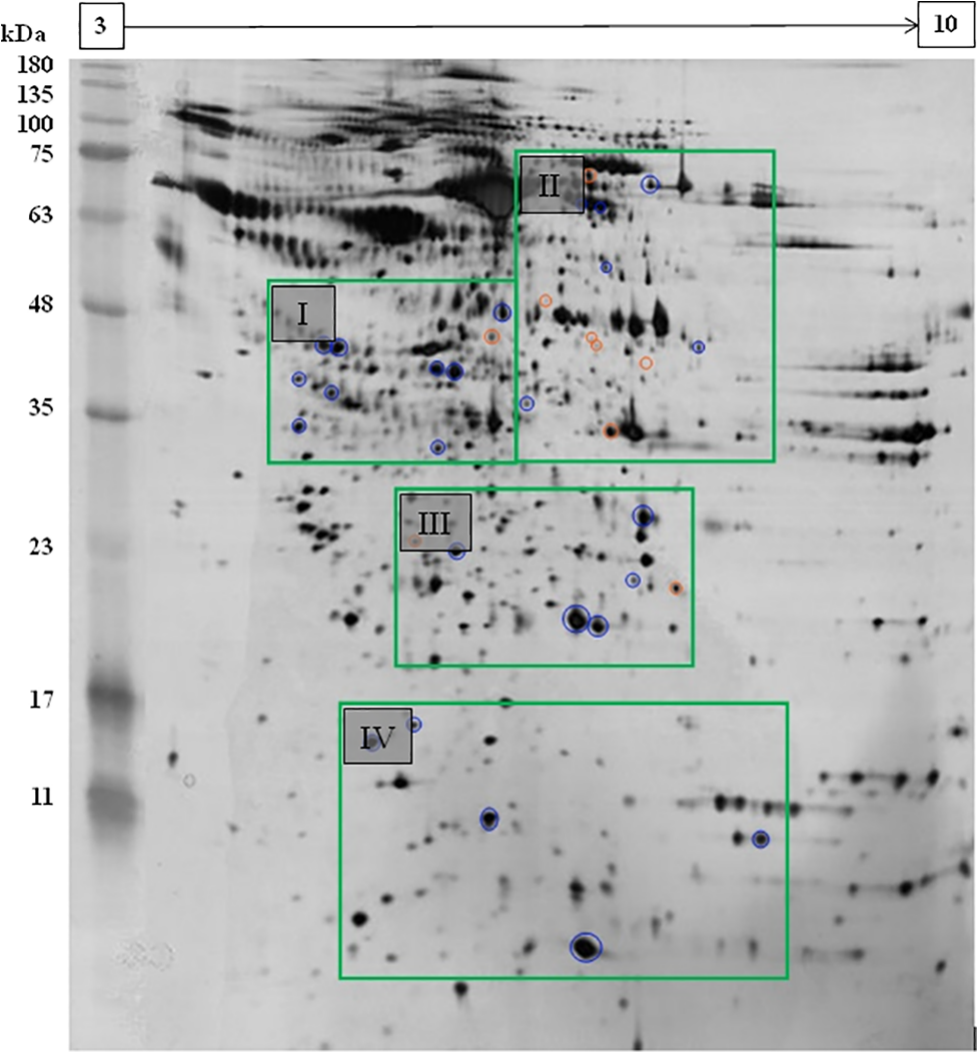
Representative secretome map of WRL-68 cells. A total of 40μg of mock control and CHIKV-infected WRL-68 secretome were resolved on 13 cm linear Immobiline DryStrip, pH 3–10 in the first dimension and 12.5% SDS-PAGE gel in the second dimension. The silver stained gels were analysed using ImageMaster gel analysis software. A total of 34 protein spots were determined to be differentially expressed, nine of which were up-regulated (circled in orange) whereas 25 were down-regulated (circled in blue). Boxed areas (I, II, III and IV) show the location of the expanded images illustrated in **Fig 3**.

**Fig 3:**
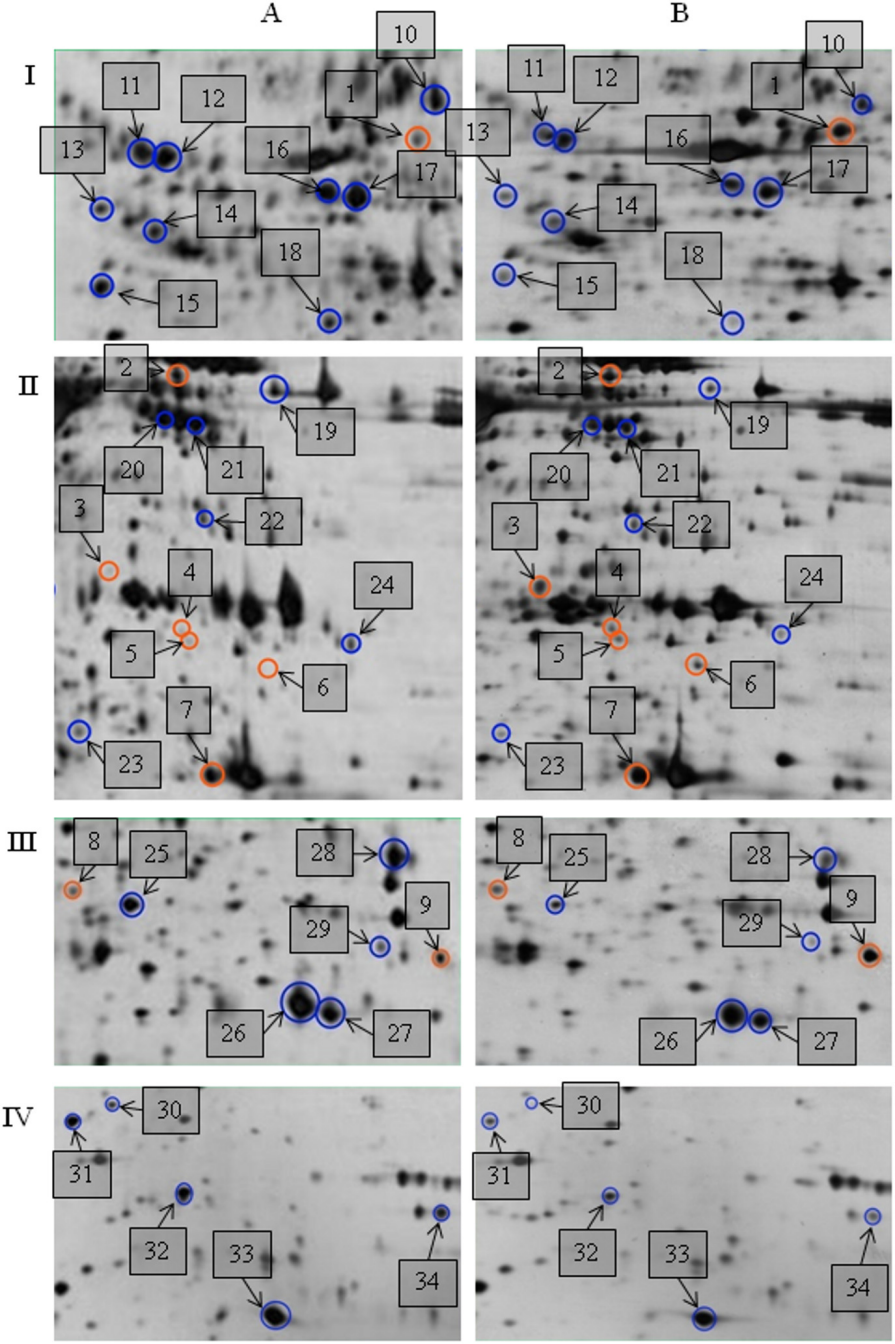
Expanded views showing the location of differentially expressed protein spots on the mock control and CHIKV-infected WRL-68 secretome gels. Expanded view of five sections—I, II, III and IV, showing differentially expressed proteins between A: mock control and B: CHIKV-infected 2DE gels. Spots 1 to 9 refer to up-regulated protein spots, whereas DS10 to DS34 refer to down-regulated protein spots. Five biological replicates per group (n = 5) were used in the analysis.

A representative 2DE gel of the secretome is provided in Fig 2. An expanded view showing spots with different abundance between the secretome of mock control and CHIKV infected cells is shown in Fig 3. Five biological replicates (five individual cultures) were used for analysis of each group. More than 1,300 individual protein spots were resolved by 2DE. Quantitative analysis of normalized spot volume revealed 34 spots with significant differences in abundance (at least 1.5 fold difference and *p*<0.05, as determined by ANOVA and Student’s t-test). The majority of the protein spots were found to be decreased in abundance. The identification of these proteins was then performed by MALDI-TOF MS/MS. A list of identified proteins is shown in Table 1. A major proportion were found to be involved in transport as well as in immune and defense response (25.00% for both).

**Table 1:** Proteins with different abundance in the secretome of CHIKV infected cells identified by MALDI-TOF/TOF MS.

Spot no.	Protein ID	Abbreviation	Swissprot accession number	MOWSE score (% coverage)	Experimental pI/MW (theoretical pI/MW)	Mean spot volume: MOCK-control Mean ± SD	Mean spot volume: CHIKV-infected Mean ± SD	Fold-change (*p*-value)	Fold-change (*p*-value) Label-free	Peptides matched
	***Immune and defence response***									
*10	**Cathepsin D (EC 3.4.23.5)**	**CTSD**	**P07339**	**395 (38)**	**6.10/44.5 (6.1/48.2)**	**0.1655 ± 0.028**	**0.0610 ± 0.031**	**-2.72 (0.001)**	**-5.52 (2.96E-05)**	**23**
**16**	Cathepsin L1 (EC 3.4.22.15)	CTSL1	P07711	109 (5)	5.31/37.5 (6.5/39.8)	0.1904 ± 0.048	0.1072 ± 0.027	-1.78 (0.010)		2
**17**	Cathepsin L1 (EC 3.4.22.15)	CTSL1	P07711	109 (5)	5.31/37.5 (6.6/39.8)	0.3237 ± 0.067	0.1280 ± 0.051	-2.53 (0.001)		2
*19	**Complement C3 precursor**	**C3**	**P01024**	**224 (10)**	**6.02/187.0 (7.4/73.0)**	**0.0786 ± 0.033**	**0.0245 ± 0.011**	**-3.21 (0.009)**	**-6.15 (1.22E-05)**	**28**
**33**	β-2 microglobulin	B2M	P61769	68 (8)	6.06/13.7 (6.8/7.3)	0.9748 ± 0.157	0.5797 ± 0.125	-1.68 (0.002)		1
**34**	Cystatin-3	CST3	P01034	179 (47)	9.00/15.8 (8.5/9.5)	0.1393 ± 0.025	0.0512 ± 0.013	-2.72 (0.000)		14
	***Transport proteins***									
**1**	Glutamate receptor subunit 3A precursor	GRIN3A	Q8TCU5	44 (11)	7.81/125.5 (6.0/42.2)	0.0308 ± 0.013	0.0941 ± 0.046	3.05 (0.014)		18
**8**	Ran-specific GTPase-activating protein	RANBP1	P43487	102 (19)	5.19/23.3 (5.3/23.0)	0.0243 ± 0.003	0.0438 ± 0.012	1.80 (0.011)		9
**9**	GTP-binding nuclear protein Ran	RAN	P62826	227 (51)	7.01/24.4 (7.7/22.1)	0.0594 ± 0.010	0.2185 ± 0.036	3.68 (0.000)		19
**18**	Vesicular integral-membrane protein VIP36 precursor	LMAN2	Q12907	473 (44)	6.46/40.2 (5.5/33.7)	0.0307± 0.018	0.0136 ± 0.003	-2.25 (0.050)		23
**22**	Tubulointerstitial nephritis antigen-like precursor	TINAGL1	Q9GZM7	85 (16)	6.54/52.4 (7.0/55.4)	0.0475± 0.009	0.0170 ± 0.010	-2.79 (0.001)		11
	***Cell adhesion***									
**15**	Collagen alpha-1(V) chain precursor	COL5A1	P20908	199 (9)	4.94/183.4 (4.2/34.8)	0.1119 ± 0.036	0.0356 ± 0.013	-3.14 (0.002)		25
**32**	Cadherin-2 precursor	CDH2	P19022	405 (11)	4.64/99.7 (5.9/10.4)	0.2778 ± 0.060	0.0935 ±0.042	-2.97 (0.001)		18
	***Metalloproteinases inhibitor***									
**26**	Tissue inhibitor of metalloproteinases 2	TIMP2	P16035	282 (35)	7.45/24.4 (6.7/21.3)	0.2858 ± 0.105	0.1357 ± 0.059	-2.11 (0.024)		15
**27**	Tissue inhibitor of metalloproteinases 2	TIMP2	P16035	360 (39)	7.45/24.4 (7.0/21.3)	0.7474 ± 0.105	0.4013 ± 0.140	-1.86 (0.002)		20
**28**	Tissue inhibitor of metalloproteinases 1	TIMP1	P01033	498 (67)	8.46/23.2 (7.4/23.7)	0.2818 ± 0.079	0.0817 ± 0.033	-3.45 (0.001)		18
	***Carbohydrate and lipid metabolism***									
**7**	Aldose reductase (EC 1.1.1.21)	AKR1B1	P15121	212 (45)	6.51/35.8 (7.1/34.5)	0.1718 ± 0.051	0.3167 ± 0.050	1.84 (0.002)		28
*20	**Kexin type 9 precursor (EC 3.4.21.-)**	**PCSK9**	**Q8NBP7**	**288 (9)**	**6.09/74.3 (6.8/66.9)**	**0.2072 ± 0.046**	**0.0482 ± 0.020**	**-4.30 (0.001)**	**-3.77 (8.57E-06)**	**11**
*21	**Kexin type 9 precursor (EC 3.4.21.-)**	**PCSK9**	**Q8NBP7**	**67 (6)**	**6.09/74.3 (7.0/66.5)**	**0.0336 ± 0.015**	**N/A**	**N/A (0.001)**		**6**
*30	**Kexin type 9 precursor (EC 3.4.21.-)**	**PCSK9**	**Q8NBP7**	**428 (34)**	**6.09/74.3 (5.3/16.2)**	**0.1723 ± 0.045**	**0.0493 ± 0.027**	**-3.50 (0.002)**		**29**
*31	**Kexin type 9 precursor (EC 3.4.21.-)**	**PCSK9**	**Q8NBP7**	**460 (19)**	**6.09/74.3 (4.9/13.1)**	**0.1423 ± 0.008**	**0.0599 ± 0.013**	**-2.41 (0.001)**		**22**
	***Protein repair***									
**29**	Protein-L-isoaspartate O-methyltransferase (EC 2.1.1.77)	PCMT1	P22061	98 (35)	6.70/24.6 (7.3/22.3)	0.0238 ± 0.005	0.0104 ± 0.002	-2.29 (0.002)		12
	***Regulation of MAPK cascade***									
**25**	Renin receptor precursor (ATPase H(+)-transporting lysosomal accessory protein 2)	ATP6AP2	O75787	104 (380)	5.76/39.0 (5.6/22.7)	0.1632 ± 0.064	0.0370 ± 0.015	-4.42 (0.003)		18
	***Miscellaneous***									
*2	**Moesin**	**MSN**	**P26038**	**86 (22)**	**6.08/67.8 (6.9/74.5)**	**0.0829 ± 0.025**	**0.1272 ± 0.022**	**1.53 (0.021)**	**1.53 (2.47E-03)**	**22**
**24**	Plasminogen activator inhibitor 1 precursor	SERPINE1	P05121	88 (23)	6.68/45.0 (7.9/42.1)	0.0294 ± 0.010	0.0120 ± 0.005	-2.46 (0.009)		14

All identified proteins have significant MOWSE scores (*p* < 0.05) of at least 55 and are categorised according to their respective biological functions in the cells.

The expressions of four proteins (marked with * and in bold) were validated by label free LCMS/MS quantification.

A total 25 spots were successfully identified by tandem mass spectrometry (73.5% identified). These proteins were categorised based on their respective functions with information obtained from Uniprot (http://www.uniprot.org/) Knowledgebase. The identities of nine spots were not determined (3, 4, 5, 6, 11, 12, 13, 14 and 23). In addition, a number of spots were identified as the same protein. Thus, the 25 successfully identified spots corresponded to 20 proteins. The numbers in Table 1 correspond to the spot numbers in Fig 3.

To be able to perform an accurate analysis of the secretome, it was necessary to identify genuine secreted proteins and distinguish them from intracellular proteins originating from lysed, dead cells. Thus, *in silico* analysis was performed on the 20 proteins (representing 25 protein spots) that showed different expression profiles to determine protein localization and function. Our analysis showed that all proteins, with the exception of aldose reductase were predicted to be secreted via classical or non-classical mechanisms. The results are shown in Table 2.

**Table 2:** Results of *in silico* analysis using SignalP, SecretomeP and TMHMM servers predicting the localization of identified proteins.

Spot Number	Protein Name	Swissprot accession number	SignalP^a)^	SecretomeP^b)^	TMHMM^c)^
**10**	Cathepsin D(EC 3.4.23.5)	P07339	**0.781**	**0.758**	12.287
**16 & 17**	Cathepsin L1(EC 3.4.22.15)	P07711	**0.754**	**0.514**	2.569
**19**	Complement C3 precursor	P01024	**0.905**	**0.618**	8.212
**33**	β-2 microglobulin	P61769	**0.773**	**0.907**	2.525
**34**	Cystatin-3	P01034	**0.898**	**0.937**	17.968
**1**	Glutamate receptor subunit 3A precursor	Q8TCU5	**0.748**	**0.660**	**88.817**
**8**	Ran-specific GTPase-activating protein	P43487	0.102	**0.602**	0.000
**9**	GTP-binding nuclear protein Ran	P62826	0.115	**0.582**	0.017
**18**	Vesicular integral-membrane protein VIP36 precursor	Q12907	**0.662**	0.222	**25.054**
**22**	Tubulointerstitial nephritis antigen-like precursor	Q9GZM7	**0.792**	**0.935**	1.618
**15**	Collagen alpha-1(V) chain precursor	P20908	**0.783**	0.053	5.377
**32**	Cadherin-2 precursor	P19022	**0.746**	0.203	**23.346**
**26 & 27**	Tissue inhibitor of metalloproteinases 2	P16035	**0.938**	**0.854**	0.246
**28**	Tissue inhibitor of metalloproteinases 1	P01033	**0.923**	**0.765**	0.510
^**#**^ **7**	Aldose reductase(EC 1.1.1.21)	P15121	0.184	0.395	0.001
**20, 21, 30 & 31**	Kexin type 9 precursor(EC 3.4.21.-)	Q8NBP7	**0.935**	**0.761**	16.080
**29**	Protein-L-isoaspartate O-methyltransferase (EC 2.1.1.77)	P22061	0.111	**0.515**	0.043
**25**	Renin receptor precursor (ATPase H(+)-transporting lysosomal accessory protein 2)	O75787	**0.919**	**0.772**	**30.116**
**2**	Moesin	P26038	0.111	**0.530**	0.001
**24**	Plasminogen activator inhibitor 1 precursor	P05121	**0.797**	**0.664**	2.528

a) The presence of signal peptides in a protein sequence was predicted using SignalP 4.0. A protein was classified as being classically secreted when it returned a D-score exceeding 0.450 (indicated by bold numbers).

b)For the prediction of proteins secreted via non-classical secretory pathways, SecretomeP 2.0 was used. Proteins win an N-N score exceeding 0.500 (indicated by bold numbers) were predicted to be secreted.

c)Prediction of transmembrane helixes was performed using TMHMM v2.0. A protein with its expected number of amino acids in transmembrane helixes (ExpAA) exceeding 18.000 (indicated by bold numbers) was predicted to be a plasma protein.

To be classified as “secereted”, a protein must fulfil at least one of the following criteria: It is predicted by SignalP to possess a signal peptide, it is predicted by SecretomeP to non-classically secreted, or, TMHMM predicts that it possesses a transmembrane helix. All proteins, with the exception of aldose reductase (marked with a ^#^), were found to fulfil at least one of these criteria.

#### Protein network analysis

**Fig 4:**
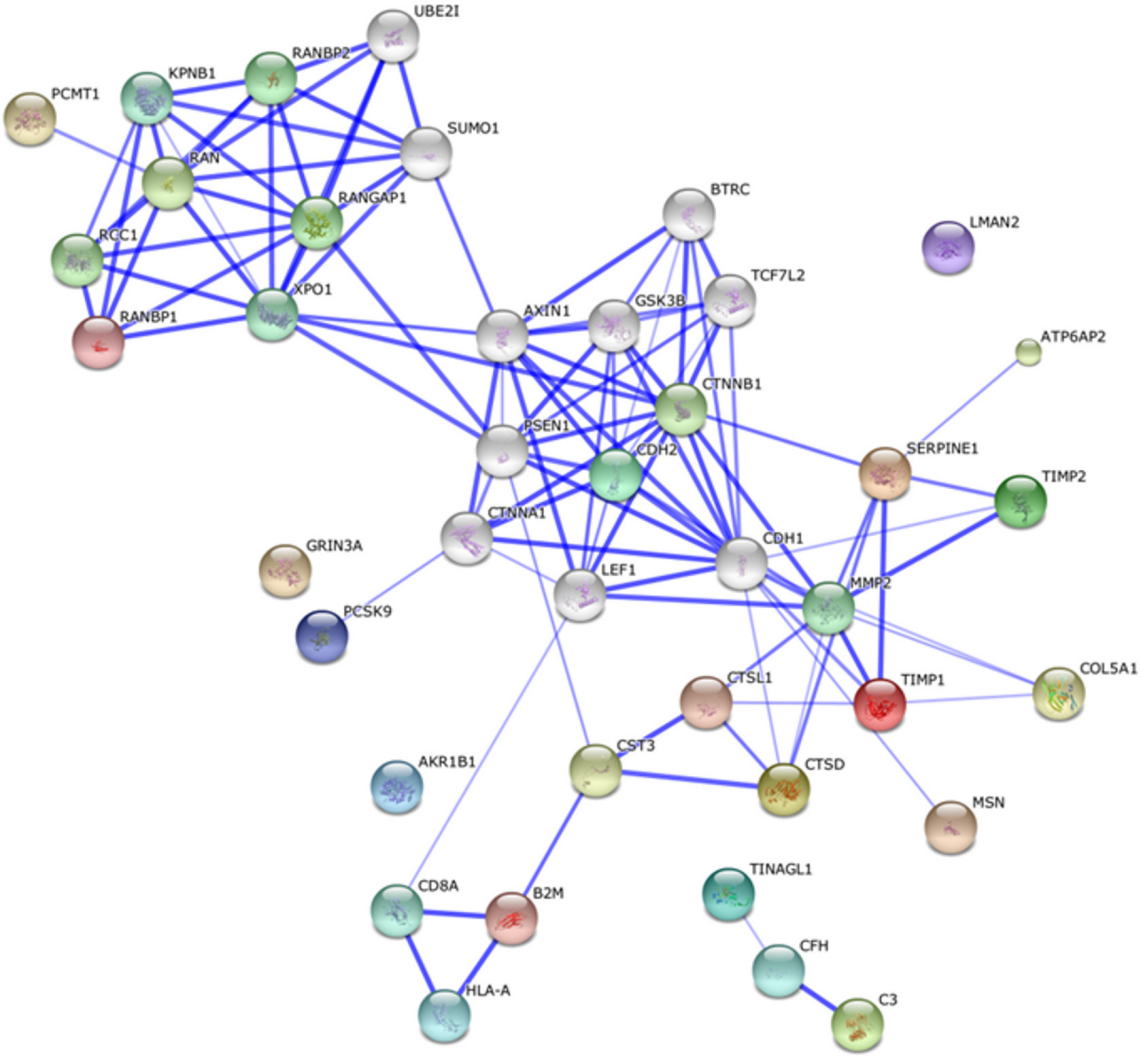
Secretome networks showing predicted functional linkages between identified proteins. STRING interaction maps were generated using default settings (Medium confidence of 0.4 and 7 criteria for linkage: neighbourhood, gene fusion, co-occurrence, co-expression, experimental evidences, existing databases and textmining). Twenty additional interplay proteins were also added to each network.

STRING network analysis of the 20 proteins with different abundance in the secretome of infected cells predicted that 17 were connected by at least one criterion (Fig 4). Gene ontology (GO) enrichment analysis of biological process was further evaluated using the STRING database to identify significant biological processes associated with the network. False discovery rate (FDR) correction was the default statistical method used to determine the significance of each process. Five processes were found to be significant in the predicted secretome network Table 3. Signal transduction, cellular component and extracellular matrix (ECM) organization, regulation of cytokine stimulus and immune response were shown to be involved in the secretome network.

**Table 3:** GO enrichment analysis of biological processes involved in the secretome.

GO Biological process	*p*-value
Regulation of cellular component organisation	7.03 x 10^−3^
Response to cytokine stimulus	1.11 x 10^−2^
Regulation of signal transduction	1.24 x 10^−2^
ECM organisation	1.70 x 10^−2^
Regulation of immune process	3.53 x 10^−2^

The significance of the GO biological processes was determined by FDR analysis (*p*< 0.05).

#### Western blot analysis

**Fig 5:**
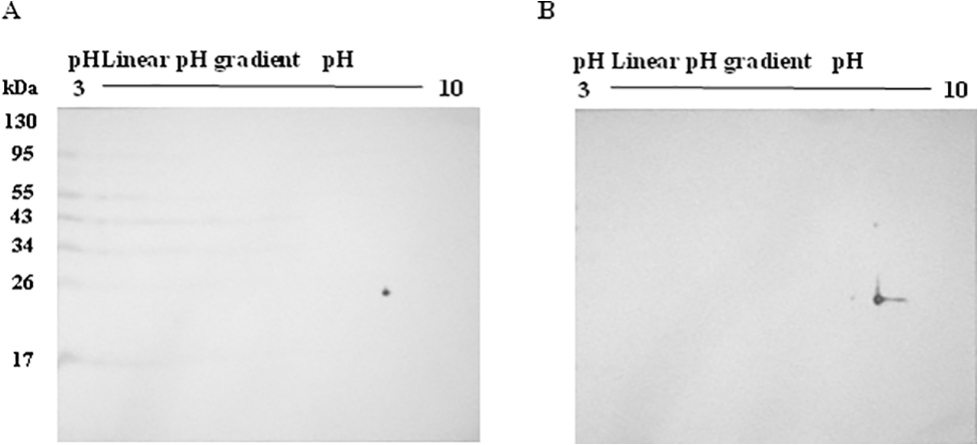
Western blot validation of protein expression for RAN. The position of the RAN protein spot for the secretome of mock control (A) and CHIKV-infected (B) WRL68 cells on 2DE immunoblots. An increase in spot intensity was observed in the CHIKV-infected blot as compared to the mock control blot.

2DE Western blot was used to ‘validate’ the position of RAN, which was identified to be increased in the secretome. Although the protein expression level cannot be accurately determined, 2DE Western blot analysis allowed confirmation of protein location on the gel based on molecular weight and pI/pH values. Fig 5 shows the position and spot intensity of RAN protein in mock control (A) and CHIKV-infected (B) 2-D immunoblots. Comparison between the immunoblots showed a similar increase in spot intensity upon CHIKV infection, as was observed with 2DE analysis.

#### Transcript expression analysis

The mRNA expression levels of 15 secreted proteins that showed different abundance, representing all functional classes, were evaluated. The transcript expression for all targeted proteins were normalised against ACTB. The results are shown in Table 4. Only one of the 15 selected proteins from the secretome sample—kexin type 9 precursor (PCSK9)—showed significant change at the mRNA expression level that was in concordance with the direction of fold change of the protein as observed in 2DE.

**Table 4:** Comparison of real-time qPCR and proteomics results for 15 selected proteins from the secretome sample.

Gene name	mRNA fold-change (*p*-value)	Protein fold-change
ATP6AP2	NSD	-4.42
B2M	NSD	-1.68
C3	NSD	-3.21
CDH2	NSD	-2.97
CST3	NSD	-2.72
CTSD	NSD	-2.72
CSTL1	NSD	-2.53, -1.78**
COL5A1	NSD	-3.14
PCMT1	NSD	-2.29
**PCSK9**	**-1.43 (0.0002)**	**-4.30, -3.50, -2.40** **
RAN	NSD	3.68
RANBP	NSD	1.80
SERPINE1	NSD	-2.46
TIMP1	NSD	-3.45
TIMP2	NSD	-2.11, -1.86**

RNA expression with similar direction of change to protein expression in 2DE is shown in **Bold (** *p*< 0.05). NSD: no significant differences in the RNA expression.

** More than one protein spot was identified.

#### Validation of protein expression by label free quantification

The expressions of four secreted proteins–cathepsin D, complement C3 precursor, kexin type 9 precursor and moesin—were validated by label free quantification. These proteins are highlighted in Table 1 (shaded in grey).

## Discussions

### Preparation of the secretome and comparative 2DE

Prior to 2DE analysis, optimisation of the MOI and time-point was performed as previously described [5] to determine suitable conditions that define early infection (the stage before cell death). This would ensure that the observed changes in the secretome can be solely attributed to CHIKV infection, and not due to other factors such as cell death [17]. It was also imperative that cell death be minimized to prevent as much as possible contamination of the secretome with cellular debris and intracellular proteins released due to cell lysis. Hence, the MOI and time-point selected for infection should induce minimal cell death as compared to the mock control, while simultaneously maintaining a significant percentage of infection. A combination of morphological analysis, IIFA and flow cytometric quantification was performed to thoroughly investigate the best condition for early infection, which resulted in the selection of the MOI of 5.0 and 24 hours post-infection incubation.

The secretome is a complex set of molecules secreted by living cells. In cell culture, these proteins are secreted into the culture media, which itself, is typically rich in a variety of proteins (due to the presence of serum which provide nutrients for cell growth). To eliminate interfering proteins in the media, serum-free DMEM medium was used during incubation following infection. However, serum starvation may have negative effects on cell growth and proliferation [18]. In addition, we have also previously shown that a slightly higher percentage of cell death in CHIKV-infected cells than in mock control cells at the selected condition for infection [5]. Therefore, the viability and integrity of cells cultured in serum-free medium was evaluated. It was also pertinent to determine that membrane integrity of CHIKV-infected cells was not greatly compromised to avoid severe contamination of the secretome with intracellular proteins.

The viability and integrity of WRL-68 cells under serum starvation and CHIKV-infection was evaluated by MTS cell proliferation and dead-cell protease assays. MTS assay measures cell viability through the activity of enzymes involved in the cytochrome system of viable cells, which reduces the MTS (a tetrazolium salt) to water-soluble formazans [19]. On the other hand, cell-death protease assay is a luminescent assay which evaluates membrane integrity by measuring the activity of proteases that have been released from dead cells. The luminogenic peptide substrate or AAF-Glo substrate (alanyl-alanyl-phenylalanyl aminoluciferin) is not permeable to viable cells with intact membranes, and therefore can only detect proteases that have been leaked out from damaged membranes [20]. The MTS assay results showed that serum starvation did not affect the viability of WRL-68 cells at 24 hours incubation. However, cell lysis was found to be inevitable, as proteases were detected even in the supernatant of control cells cultured with serum-supplemented medium. Nevertheless, the degree of cellular disruption between cells grown in serum-supplemented medium, serum-free medium and CHIKV-infected cells (MOI 5.0) incubated in serum-free medium were comparable. Taken together, it can be deduced that serum-starvation and infection at the selected MOI and time-point did not result in significant loss of cell viability and integrity.

Proteomics analysis was subsequently performed to compare the secretome profiles between mock control and CHIKV-infected WRL-68 cells. More than 1000 spots were detected, of which 34 were significantly different by at least 1.5 fold. Out of fifteen proteins selected for analysis on Transcript level, only PCSK9 showed a positive correlation with its mRNA expression while the rest were not significantly changed. This was not entirely unexpected, given that post-transcriptional modifications such as mRNA splicing or editing and post-translational modifications often determine the final translation rate of a protein [21]. It must also be noted that the localization of the identified proteins may also affect their apparent abundance in the secretome, as these proteins may be present in other localities within the cell. The poor correlation between the transcript and secretome expression levels also suggested that other mechanisms may be involved in determining the protein secretion rate, such as protein folding and cell cycle position [22, 23].

The focus of this study was to understand the dynamic interplay of differentially expressed secretome across subcellular fractions in detail as characterization of these proteins at total proteome level is difficult. Our data showed that early CHIKV infection was found to cause widespread alterations of the secretome. Proteins of diverse functions and sub-cellular locations were found to have changed in abundance with a majority of them being decreased. Only one protein identified in the secretome (aldose reductase) was not predicted to be a secreted protein demonstrating the robustness of our secretome preparation. In terms of functional classification, a large subset of these proteins were found to be involved in metabolism, DNA/RNA/protein biogenesis, immune response and transport, suggesting that these functions may be important during CHIKV infection in host cells.

STRING network analysis was used to predict the functional interactions between proteins based on existing information from public text collections and experimental data. Three immune and defense response proteins; cystatin 3 (CST3), cathepsin D (CTSD) and cathepsin L1 (CTSL1), showed strong linkages in the secretome network, as shown in Fig 4. Beta-2 microglobulin (B2M), on the other hand, was linked to the major histocompatibility (MHC) class I complex through human leukocyte antigen A (HLA-A). Tubulointerstitial nephritis antigen-like precursor (TINAGL1), was linked to complement proteins, complement C3 (C3) and complement factor H (CFH) in a separate cluster. Tissue inhibitor of metalloproteinase 1 and 2 (TIMP1 and TIMP2), and collagen alpha-I (V) chain precursor (COL5A1) were indirectly connected through matrix metalloproteinase 2 (MMP2) protein.

The predicted functional linkages were then used to determine significant biological processes involved in each network. The FDR-filtered (*p* < 0.05) list of biological processes showed that proteins that governed immune response, as well as ECM and cellular compartment organisation were identified in the secretome network. The known functions of the proteins that changed in abundance are discussed in the following sections.

### Classification of regulated proteins based on function

#### Alteration of proteins associated with cellular transport and signalling

Virus infections always involve hijacking of selected host cellular factors that are advantageous in sustaining virus survival. These factors may include proteins associated with the cellular transport system as identified in the current study where 25% of secreted proteins with different abundance were transport proteins. These proteins included—GTP-binding nuclear protein ran (RAN), ran-specific GTPase activating protein (RANBP1) and glutamate receptor subunit 3A precursor (GRIN3A).

RAN together with RANBPs and RANGAP, form a RAN GTPase system associated with energy driven activities such as cell cycle progression, protein importation and mRNA exportation [24, 25]. Rapid secretion of these nuclear proteins to the extracellular matrix may suggest an essential role in facilitating replication of positive strand RNA virus which is known to replicate exclusively in the cytoplasm.

GRIN3A is an NMDA receptor subtype of glutamate-gated ion channel with vital roles such as ionotropic glutamate receptor activity and calcium ion and magnesium ion binding. Induction of glutamate receptor following alphaviruses infection has been shown to stimulate membrane depolarization for viral entry mediated by Ca^2+^ [26].

#### Deregulation of proteins involved in cell immune and defence response

The majority of positive strand RNA viruses are programmed to target the host defence mechanism [27]. In our analysis, of the proteins in the secretome that changed in abundance during CHIKV infection, 25% were proteins involved in cell immune and defence response. These proteins included cathepsin D (CTSD), cathepsin L1(CTSL1), complement C3 precursor (C3), ß-2 microglobulin (B2M) and cystatin-3 (CST3).

Cathepsins are lysosomal proteases involved in a number of physiological processes. These processes include apoptosis, proliferation and activation of inflammation, which are processes often observed in infected cells [28, 29]. In our analysis we found two cathepsins—CTSD and CTSL1—to be decreased. Cathepsin activities are heavily dependent on modulation of their endogenous inhibitor; Cystatin-3 (CTS3) [30]. CTS3 is a secreted cysteine protease inhibitor extensively distributed in the extracellular matrix. It functions in the regulation of apoptosis and exert inhibitory effects against viral replication and RNA virus induced apoptosis [31, 32]. The observed decrease in abundance of CTS3, together with the cathepsins, may have an effect in promoting CHIKV dissemination.

#### Deregulation of proteins involved in cell adhesion

Cell adhesion is an essential structural network of intracellular system for effective immune responses and tissue repair [33]. Viruses have been shown to utilize unique strategies in exploiting cell adhesion. These strategies include those associated with viral binding, viral internalization and shunning of the host’s immune surveillance [34–36]. In virus infected cells, the loss of the cell’s adhesive characteristics is always accompanied by cell death or injury.

Our analysis showed that two cell adhesion associated proteins—collagen alpha-1 (V) chain precursor (COL5A1) and cadherin-2 precursor (CDH2)—were decreased in the secretome of CHIKV infected cells. COL5A1 is a member of collagen that regulates fibril formation which with its deficiency will cause tissue disorders such as fragility, joint weakness and loss of tissue strength as manifested in almost all CHIK infected patients [37]. Meanwhile, CDH2 is a trans-membrane glycoprotein that heavily depends on the presence of extracellular Ca^2+^ for a stable cell to cell adhesion.

#### Perturbation of cellular energy production and host cell metabolism

In this study, two proteins identified from the secretome were involved in cellular metabolism. We have also previously shown that a further 19 proteins involved in cellular metabolism from the whole cell proteome of CHIKV infected cells changed in abundance [5]. Taken together, this suggested severe disruption of the host cell metabolism upon CHIKV infection.

Aldose reductase (AKR1B1), a catalyst involved in the reduction of glucose to sorbitol in glucose metabolism, was found to be increased by 1.84 times in the current study. Elevated AKR1B1 expression is closely related to a self defense mechanism against cell mortality triggered by nitric oxide [38]. Interestingly, *In Silico* analysis revealed that AKR1B1 does not fulfil any of the criteria outlined to qualify it as a secreted protein (Table 2).

The abundance of the protein kexin type 9 precursor, PCSK9 (4 spots) was found to have decreased in the secretome. This observation correlated with its mRNA expression level as shown through quantitative RTPCR (Table 4). Furthermore, western blotting also showed similar directionality in the expression of PCSK9 (Fig 5). PCSK9 is a known inhibitor for the low density lipoprotein receptor (LDLR), an important receptor for hepatitis C virus replication [39].

In conclusion, the current study represents a first attempt in a comprehensive comparative analysis of secretome derived from CHIKV infected cells, revealing host factors that may be involved in CHIKV infection. We showed that a majority of the affected secreted proteins were those involved in cellular transport and signaling, immune response, cell adhesion and cellular metabolism. The results obtained here will add to currently available knowledge of late host response studies. However, before we can draw concrete conclusions with regards to the involvement of these proteins in virus-host interaction, further functional characterization will need to be performed.

## Supporting Information

S1 Table Protein names and gene symbols used in the secretome network. (DOCX)
