# Supplementary material for: Differential Analysis of the Secretome of WRL68 Cells Infected with the Chikungunya Virus
Source: PLoS One. 2015 Jun 17;10(6):e0129033. doi: 10.1371/journal.pone.0129033 (PMC4470940; doi:10.1371/journal.pone.0129033)
Supplement: S1 Table — (DOCX) [file pone.0129033.s001.docx]

**Table S1: Protein names and gene symbols used in the secretome network**

| **Protein ID** | **Gene Name** |
| --- | --- |
| Cathepsin D | CTSD |
| Cathepsin L1 | CTSL1 |
| Complement C3 precursor | C3 |
| β-2 microglobulin | B2M |
| Cystatin-3 | CST3 |
| Glutamate receptor subunit 3A precursor | GRIN3A |
| Ran-specific GTPase-activating protein | RANBP1 |
| GTP-binding nuclear protein Ran | RAN |
| Vesicular integral-membrane protein VIP36 precursor | LMAN2 |
| Tubulointerstitial nephritis antigen-like precursor | TINAGL1 |
| Collagen alpha-1(V) chain precursor | COL5A1 |
| Cadherin-2 precursor | CDH2 |
| Tissue inhibitor of metalloproteinases 2 (TIMP-2) | TIMP2 |
| Tissue inhibitor of metalloproteinases 1 (TIMP-1) | TIMP1 |
| Aldose reductase | AKR1B1 |
| Proprotein convertase subtilisin/kexin type 9 precursor | PCSK9 |
| Protein-L-isoaspartate O-methyltransferase | PCMT1 |
| Renin receptor precursor (ATPase H(+)-transporting lysosomal accessory protein 2) | ATP6AP2 |
| Moesin | MSN |
| Plasminogen activator inhibitor 1 precursor | SERPINE1 |
| Karyopherin (Importin) beta 1 | KPNB1 |
| Regulator of chromosome condensation 1 | RCC1 |
| Exportin 1 | XPO1 |
| Ran binding protein 2 | RANBP2 |
| Ran GTPase activating protein 1 | RANGAP1 |
| SUMO1 pseudogene 3 | SUMO |
| Ubiquitin-conjugating enzyme E2I | UBE2I |
| Axin 1 | AXIN1 |
| Glycogen synthase kinase 3 beta | GSK3B |
| Beta transducin repeat containing | BTRC |
| Transcription factor 7-like 2 | TCF7L2 |
| Catenin alpha 1 | CTNNA1 |
| Catenin beta 1 | CTNNB1 |
| Presenilin 1 | PSEN1 |
| Cadherin 1 | CDH1 |
| Lymphoid enhancer-binding factor 1 | LEF1 |
| CD8a molecule | CD8A |
| Matrix metalloproteinase 2 | MMP2 |
| Major histocompatibility complex , class I, A | HLA-A |
| Complement factor H | CFH |
